# Transcriptional repression and DNA hypermethylation of a small set of ES cell marker genes in male germline stem cells

**DOI:** 10.1186/1471-213X-6-34

**Published:** 2006-07-21

**Authors:** Masanori Imamura, Kyoko Miura, Kumiko Iwabuchi, Tomoko Ichisaka, Masato Nakagawa, Jiyoung Lee, Mito Kanatsu-Shinohara, Takashi Shinohara, Shinya Yamanaka

**Affiliations:** 1 Department of Stem Cell Biology, Institute for Frontier Medical Sciences, Kyoto University, Kyoto 606-8507, Japan; 2 Department of Molecular Genetics, Graduate School of Medicine, Kyoto University, Kyoto 606-8507, Japan; 3 Horizontal Medical Research Organization, Graduate School of Medicine, Kyoto University, Kyoto 606-8501, Japan; 4 CREST, Japan Science and Technology Agency, Saitama, Japan

## Abstract

**Background:**

We previously identified a set of genes called ECATs (ES cell-associated transcripts) that are expressed at high levels in mouse ES cells. Here, we examine the expression and DNA methylation of ECATs in somatic cells and germ cells.

**Results:**

In all ECATs examined, the promoter region had low methylation levels in ES cells, but higher levels in somatic cells. In contrast, in spite of their lack of pluripotency, male germline stem (GS) cells expressed most ECATs and exhibited hypomethylation of ECAT promoter regions. We observed a similar hypomethylation of ECAT loci in adult testis and isolated sperm. Some ECATs were even less methylated in male germ cells than in ES cells. However, a few ECATs were not expressed in GS cells, and most of them targets of Oct3/4 and Sox2. The Octamer/Sox regulatory elements were hypermethylated in these genes. In addition, we found that GS cells express little Sox2 protein and low Oct3/4 protein despite abundant expression of their transcripts.

**Conclusion:**

Our results suggest that DNA hypermethylation and transcriptional repression of a small set of ECATs, together with post-transcriptional repression of Oct3/4 and Sox2, contribute to the loss of pluripotency in male germ cells.

## Background

Embryonic stem (ES) cells possess many unique properties, including long-term self-renewal and pluripotency, which is the ability to differentiate into all types of somatic and germ cells[1,2]. Previous studies showed that pluriopotency in ES cells and early embryos depend on genes that are specifically expressed in pluripotent cells. These genes, collectively dubbed "ECATs" for ES cell associated transcripts, include transcription factors such as Oct3/4 and Sox2. Oct3/4 maintains ES cells in an undifferentiated state in a dose-dependent manner[3,4], and Sox2 functions synergistically with Oct3/4 in this process[5].

In addition to *Oct3/4 *and *Sox2*, we have identified a number of novel ECATs using digital differential display of expressed sequence tag (EST) databases. We found that *Nanog*/*ecat4 *is a homeodomain protein essential for self-renewal and pluripotency in ES cells and early embryos. Overexpression of *Nanog *allows for sustained self-renewal of ES cells even in the absence of leukemia inhibitory factor (LIF)[6,7]. Another ECAT member, *ERas*/*ecat5*, is a constitutively active Ras-like protein that promotes the robust proliferation of ES cells[8].

Two possible mechanisms could account for the ES cell-specific expression of ECATs. One is the ES cell-specific expression of transcription factors that regulate expression of downstream ECATs. An example of this sort of *trans*-acting regulation is the activation of ES cell-specific genes such as *Fgf4*[9], *Rex1*[10], *Utf1*[11], *Fbx15*[12], and *Nanog *[13-15] by Oct3/4 and Sox2, which can also activate their own expression [16-18]. Alternatively, ES cell-specific expression could be achieved by epigenetic modifications, such as DNA methylation. For example, the *cis*-acting promoter and proximal/distal enhancer regions of *Oct3/4 *are hypomethylated in ES cells, whereas they are heavily methylated in somatic cells and in trophectoderm lineages[19]. Deletion of *Dnmt3a *and *Dnmt3b*, which are *de novo *DNA methyltransferases, results in global hypomethylation of genomic DNA and partial resistance to differentiation in mouse ES cells[20]. A similar phenomenon was also observed when ES cells were deprived of *CpG binding protein*[21]. These findings indicate that DNA methylation plays a pivotal role in gene regulation during differentiation and development.

Germ cells are themselves neither pluripotent nor totipotent, but are able to transmit totipotency to the next generation. The rapid recovery of totipotency by germ cells upon fertilization stands in stark contrast to the inability of somatic cells to recover totipotency or pluripotency once they have differentiated. Since ECATs play important roles in totipotency and pluripotency, it is possible that they are differentially regulated in somatic cells and germ cells. To test this idea, we examined the expression and DNA methylation of ECATs in somatic cells and germ cells. We found that many ECATs, including *Oct3/4 *and *Sox2*, were expressed in male germline stem (GS) cells, which are cultured spermatogonial stem cells derived from newborn mouse testes[22], despite their highly restricted potential. Furthermore, the regulatory regions of these genes were hypomethylated in GS cells and mature sperm. However, some ECAT genes, including *Nanog*, *ECAT1*, *Fbx15*, and *Fgf4*, were not expressed in GS cells. Among these, *Nanog*, *Fbx15*, and *Fgf4 *have been shown to be direct targets of synergistic activation by Oct3/4 and Sox2. The Octamer motif and Sox-binding sites of these three genes were hypermethylated in GS cells. Unexpectedly, we found that GS cells showed low Oct3/4 and little Sox2 protein levels despite high expression levels of the corresponding mRNA. We argue that the repression and DNA hypermethylation of a small set of ECATs, and the post-transcriptional suppression of Oct3/4 and Sox2 contribute to the loss of pluripotency in male germ cells and the rapid recovery of totipotency following fertilization.

## Results

### Most ECATs are expressed in male germline stem cells

**Figure 1:**
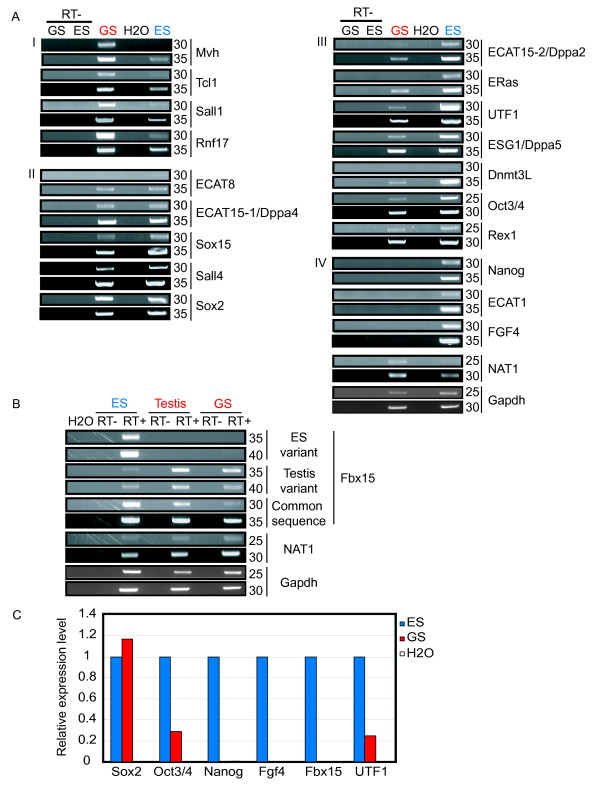
**Expression of ECATs in male germline stem (GS)cells and ES cells**. A. RT-PCR was performed for the number of cycles shown on the right. We categorized ECATs into four groups based on their relative expression levels in GS cells compared to ES cells. To monitor amplification from genomic DNA, we amplified samples in which reverse transcriptase was omitted from the reverse transcription reaction (RT-). As negative controls, water was added instead of cDNA (H2O). *NAT1 *and *Gapdh *were used as loading controls. B. Expression of *Fbx15 *variants examined by RT-PCR. Our previous study using 5' RACE showed that *Fbx15 *is transcribed from different promoters in ES cells and testes in the mouse (unpublished data). The ES cell-specific variant was not detected in GS cells or adult testis, while the testis-specific variant was weakly detected in ES cells. The two transcripts differ only in exon 1 sequence. The common sequence of the two transcripts was also amplified. *NAT1 *and *Gapdh *were used as loading controls.C. Real-time PCR quantification of the expression of Oct3/4 and Sox2 target genes. The expression of each gene was normalized with that of *Gapdh*. The expression in ES cells was set as 1.0.

To examine the expression of ECAT genes in germ cells, we performed RT-PCR analysis (Fig. 1). Expression of the germline marker *mouse vasa homolog *(*Mvh*)[23] confirmed GS cell identity. Most ECAT genes were expressed in GS cells but at different levels than in ES cells. *Stella/dppa3 *(Fig. 8), *Tcl1*, *Sall1*, and *Rnf17 *were expressed at higher levels in GS cells than in ES cells (group I), while GS cells and ES cells expressed similar levels of *ECAT8*, *ECAT15-1/Dppa4*, *Sox15*, *Sall4*, and *Sox2 *(group II). *ECAT15-2/Dppa2*, *ERas*, *Gdf3 *(Fig. 8), *Utf1*, *Esg1/Dppa5*, *Dnmt3L, Oct3/4*, *and Rex1 *expression was detected in GS cells, but at lower levels than in ES cells (group III).

### Repression of a small set of ECATs in GS cells

Although most ECATs were expressed in GS cells, we could not detect expression of *ECAT1*, *Fgf4*, or *Nanog *(group IV). In addition, we discovered that the ES cell-specific variant of *Fbx15 *was expressed in ES cells, but not in GS cells or testis. In contrast, the testis-specific variant of *Fbx1*5, which is transcribed from a different promoter, is expressed at high levels in GS cells and testis and but only weakly in ES cells (Fig. 1B). Quantification of transcript levels by real-time PCR confirmed that expression of *Nanog*, *Fgf4*, and *Fbx15 *was lost in GS cells (Fig. 1C).

### DNA hypomethylation of ECATs that are expressed in GS cells

**Figure 2:**
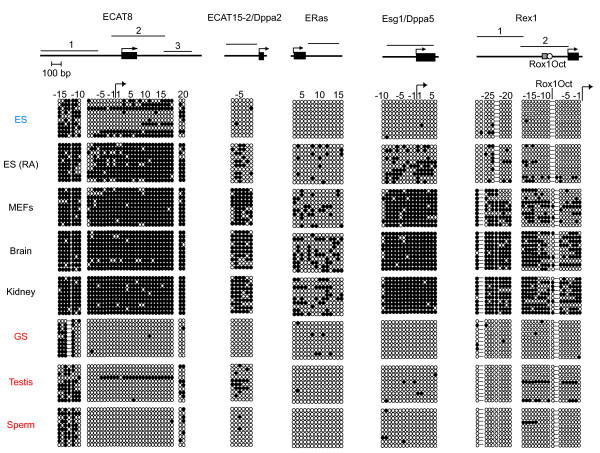
**DNA methylation states of ECATs expressed in GS cells**. Bisulfite genomic sequencing was performed to examine the DNA methylation profiles for the individual CpG sites of ECAT genes in ES cells, somatic cells, and male germ cells. Schematic diagrams of regions analyzed are shown in the upper part of the figure. Black rectangles represent the exons, and arrows indicate transcription initiation sites. Black horizontal bars indicate the regions analyzed. All of diagrams are in scale relative to a 100-bp scale bar. In the diagram for the *Rex1 *gene, the shaded square and open circle indicate the binding sites for Rox1 and Oct3/4, respectively. DNA methylation states in ES cells, somatic cells, and germ cells are shown in the lower part of the figure. Open circles indicate unmethylated CpGs and closed circles indicate methylated CpGs. The numbers indicate the relative positions of CpG sites from the transcription initiation site. The CpG nearest to the transcription initiation site is described as -1 (upstream) or 1 (downstream). Positions where CpG is absent due to DNA polymorphism are indicated by hyphens. ES, undifferentiated ES cells (129 background); ES (RA), ES cells differentiated by retinoic acid treatment; MEFs, MEFs derived from E13.5 C57BL/6J embryos; GS, GS cells derived from DBA neonate testis. Brain, kidney, and testis were obtained from 8-week-old C57BL/6J mice. Sperm was isolated from the epididymis of 11-week-old C57BL/6J mice.

The expression of ECAT genes in GS cells suggested that they might show similar DNA methylation patterns in ES cells. To test this possibility, we performed bisulfite genomic sequencing of the regulatory regions of *ECAT8*, *ECAT15-2*/*Dppa2*, *ERas*, *Esg1*/*Dppa5*, and *Rex1 *(Fig. 2), all of which were expressed in both ES cells and GS cells. In ES cells, *ECAT15-2*/*Dppa2*, *ERas*, *Esg1*/*Dppa5*, and *Rex1 *showed hypomethylation, whereas *ECAT8 *showed partial methylation. In GS cells, *ECAT15-2*/*Dppa2*, *ERas*, *Esg1*/*Dppa5*, and *Rex1 *showed similar hypomethylation to that observed in ES cells. *ECAT8 *had lower methylation in GS cells than in ES cells. Similar methylation states were observed in testis and sperm from adult mice. We also observed hypomethylation of the promoters of *Gdf3 *and *Stella/Dppa3 *in GS cells (Fig. 9).

In contrast, all examined genes were methylated in somatic cells, but to varying degrees. *ECAT8*, *ECAT15-2*/*Dppa2*, and *Esg1*/*Dppa5 *were heavily methylated, while *Rex1 *was ~70% methylated. The intronic region flanking the 3'-end of the first exon of *ERas *was methylated in somatic cells, but to a much lesser degree than the *Esg1/Dppa5 *and *ECAT15-2*/*Dppa2 *promoters. We found that for each gene, there were no significant differences in methylation patterns between mouse embryonic fibroblasts, adult kidney, and brain. Retinoic acid-treated ES cells exhibited methylation patterns that were intermediate between those of ES cells and somatic cells.

**Figure 3:**
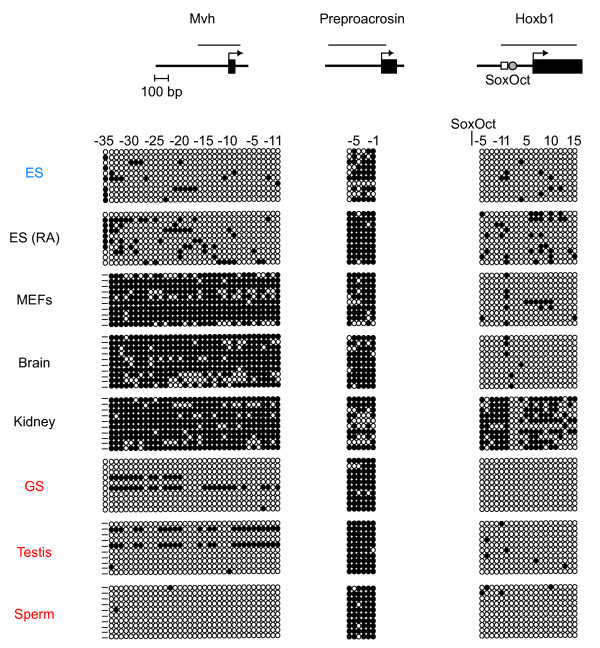
**DNA methylation states of non-ECAT genes**. Bisulfite genomic sequencing was performed to examine the DNA methylation profiles for individual CpG sites in the promoter regions of germ cell specific genes *Mvh *and *preproacrosin*, and a differentiation marker *Hoxb1 *in ES cells, somatic cells, and male germ cells. Results are shown as described in Figure 2. In the diagram for the *Hoxb1 *gene, the open square and shaded circle indicate the binding sites for Sox2 and Oct1, respectively.

In addition to the ECAT genes, we examined the DNA methylation status of germ cell specific genes *Mvh *and *preproacrosin*, and a differentiation marker *Hoxb1 *(Fig. 3). The *Mvh *promoter exhibited a methylation pattern similar to those of the ECAT genes. The *preproacrosin *promoter was largely methylated in all cells and tissues tested. In contrast, the *Hoxb1 *promoter was unmethylated in most samples except for kidney.

### Oct3/4- and Sox2-binding sites of some ECATs are hypermethylated in male germ cells

**Figure 4:**
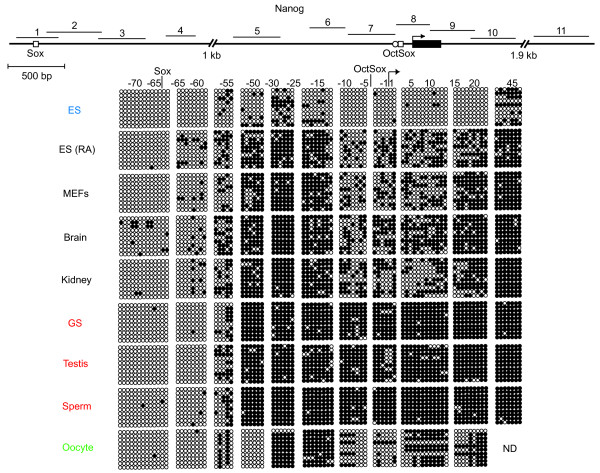
**DNA methylation state of the *Nanog *locus**. Bisulfite genomic sequencing was performed to examine the DNA methylation profile of individual CpG sites in the regions flanking exon 1 of *Nanog *in ES cells, somatic cells, and male and female germ cells. Results are shown as described in Figure 2 and 3 except for the size of the scale bar (500 bp). Oblique lines indicate regions not examined and their approximate lengths are shown. Oocytes were collected from oviducts of C57BL6 mice after superovulation. ND; not determined.

Next, we examined the DNA methylation state of ECAT genes that were not expressed in GS cells. We found that the proximal enhancer region of *Nanog*, which is regulated by Oct3/4 and Sox2, was hypermethylated in the male germline (Fig. 4). This region showed only partial methylation in somatic cells. In contrast, there was no difference in methylation state between male germ cells and somatic cells in regions both upstream and downstream of the *Nanog *proximal enhancer. These results indicate that hypermethylation occurs in a region-specific manner. Moreover, we found that methylation of the *Nanog *locus was lower in oocytes than in somatic cells, indicating that the methylation in germ cells occurs in a sex-specific manner.

**Figure 5:**
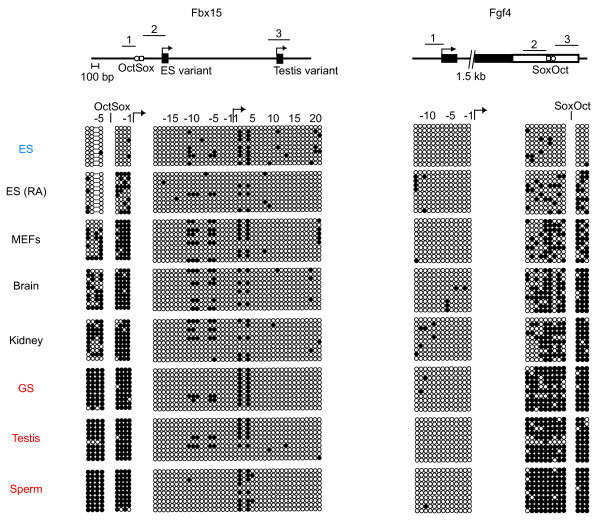
**DNA methylation states of *Fbx15 *and *Fgf4 ***. Bisulfite genomic sequencing was performed to examine the DNA methylation profiles for individual CpG sites in the *Fbx15 *and *Fgf4 *loci in ES cells, somatic cells, and male germ cells. Results are shown as described in Figure 2 and 3. Oblique lines indicate regions not examined and their approximate lengths are shown. White rectangle in the diagram for *Fgf4 *gene indicates 3' UTR.

Hypermethylation was also observed in the *Fgf4 *and *Fbx15 *enhancers (Fig. 5). The *Fgf4 *enhancer, which is regulated by Oct3/4 and Sox2, was hypermethylated in GS cells, adult testis, and isolated sperm, but was partially methylated in somatic cells, including MEFs, kidney, and brain. By contrast, the *Fgf4 *promoter was hypomethylated in both male germ cells and somatic cells. The ES cell-specific Oct/Sox-dependent enhancer of *Fbx15 *was more methylated in male germ cells than in somatic cells, whereas the testis-specific promoter was hypomethylated in all cell types analyzed.

**Figure 6:**
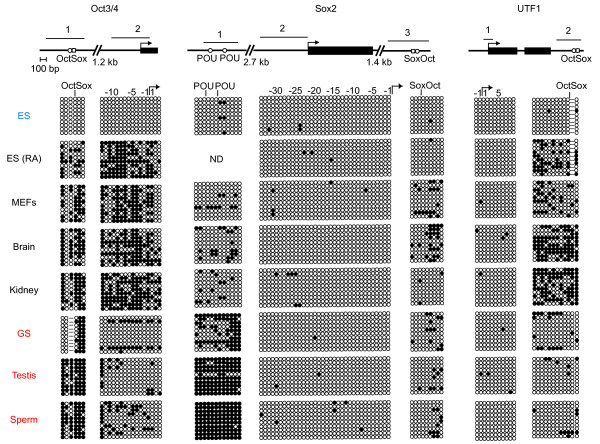
**DNA methylation states of *Oct3/4*, *Sox2*, and *Utf1 ***. Bisulfite genomic sequencing was performed to examine the DNA methylation profiles for individual CpG sites in the *Oct3/4*, *Sox2*, and *Utf1 *loci in ES cells, somatic cells, and male germ cells. Results are shown as described in Figure 2 and 3. Oblique lines indicate regions not examined and their approximate lengths are shown.

These data raise the possibility that Oct3/4 and/or Sox2 binding sites might be targets of hypermethylation in male germ cells. To investigate this possibility, we examined the enhancers of *Oct3/4*, *Sox2*, *and Utf1*, which have previously been shown to be regulated by Oct3/4 and Sox2 (Fig. 6). We found that the Octamer/Sox element of *Oct3/4 *was partially methylated in GS cells but hypermethylated in testis and sperm. Similarly, the *Sox2 *upstream enhancer, which has two Octamer sites, also exhibited hypermethylation specifically in male germ cells.

**Figure 7:**
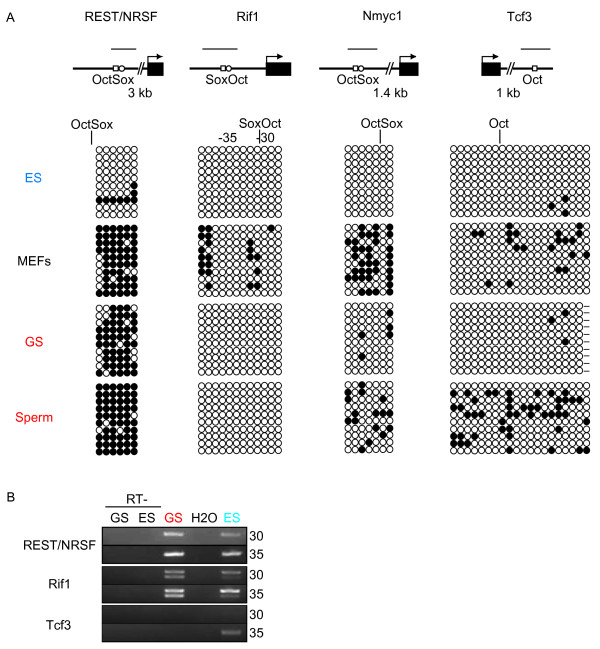
**DNA methylation states of the Octamer/Sox elements of non-ECAT genes**. A. Bisulfite genomic sequencing was performed to examine the DNA methylation profiles for individual CpG sites in the Octamer/Sox elements of non-ECAT genes in ES cells, somatic cells, and male germ cells. Results are shown as described in Figure 2 and 3. Oblique lines indicate regions not examined and their approximate lengths are shown. B. Results of RT-PCR showing the expression of *REST*/*NRSF*, *Rif1*, and *Tcf3 *in ES cells and GS cells.

However, the Oct/Sox elements of the *Sox2 *and *Utf1 *enhancers did not follow this pattern: The Oct/Sox element in *Sox2 *was hypomethylated in all cells, whereas that of *Utf1 *was partially methylated only in somatic cells. The Oct/Sox elements in non-ECAT genes *Rif1*, *Nmyc1*, and *Tcf3*[24] also did not exhibit hypermethylation in germ cells either. The Oct/Sox site of *REST*/*NRSF *[24] was hypermethylated not only in male germ cells, but also in somatic cells (Fig. 7). These data demonstrate that some, but not all, Oct/Sox elements are selectively hypermethylated in male germ cells.

**Figure 8:**
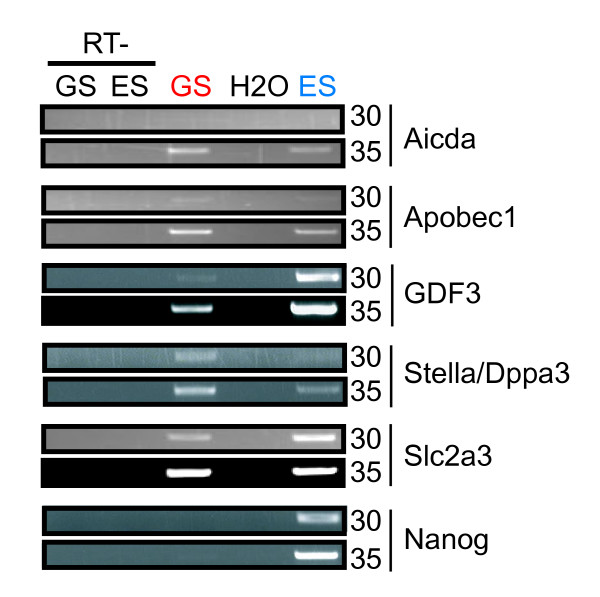
**Expression of genes located in the ECAT cluster on chromosome 6**. RT-PCR was performed to examine the expression of *Aicda*, *Apobec1*, *Gdf3*, *Stella*, *Slc2a3*, and *Nanog*. PCR amplification cycle numbers are shown on the right.

**Figure 9:**
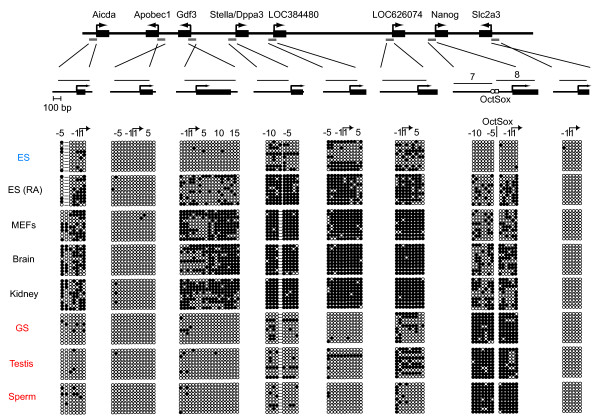
**DNA methylation states of genes in the ECAT cluster on chromosome 6**. Bisulfite genomic sequencing was performed to examine the DNA methylation profile of individual CpG sites in the promoter regions of *Aicda*, *Apobec1*, *Gdf3*, *Stella*/*Dppa3*, *LOC384480*, *LOC626074*, *Nanog*, and *Slc2a3*. Results are shown as described in Figure 2 and 3.

### Methylation state of the ECAT cluster on mouse chromosome 6

Three ECATs, *Gdf3*, *Stella*, and *Nanog*, are clustered on chromosome 6 in the mouse and chromosome 12 in humans. This region contains other genes, including *Aicda*, *Apobec1*, *LOC384480*, *LOC626074*, and *Slc2a3 *in the mouse. We performed RT-PCR and found that *Aicda*, *Apobec1*, and *Slc2a3 *are expressed in both GS cells and ES cells (Fig. 8). Since *LOC384480 *and *LOC626074 *both encode ribosomal proteins that share similar sequences with numerous different genes, we were unable to specifically amplify these two sequences and therefore could not study their expression in ES cells and GS cells. Nevertheless, our data indicate that this region is transcriptionally active in both ES cells and GS cells, with the exception of *Nanog*, which is not expressed in GS cells.

We next studied the methylation state of this region (Fig. 9). We found that the male germ cell-specific hypermethylation was specific to *Nanog *in this region. The other genes examined were hypomethylated in GS cells, testis, and sperm. By contrast, all the genes in this region were common in low methylation levels in ES cells. Most of them were more methylated in somatic cells, except for *Apobec1 *and *Slc2a3 *that showed hypomethylation in all tissues and cells examined.

### The Oct/Sox sites are not occupied with Oct3/4 and Sox2 in GS cells

**Figure 10:**
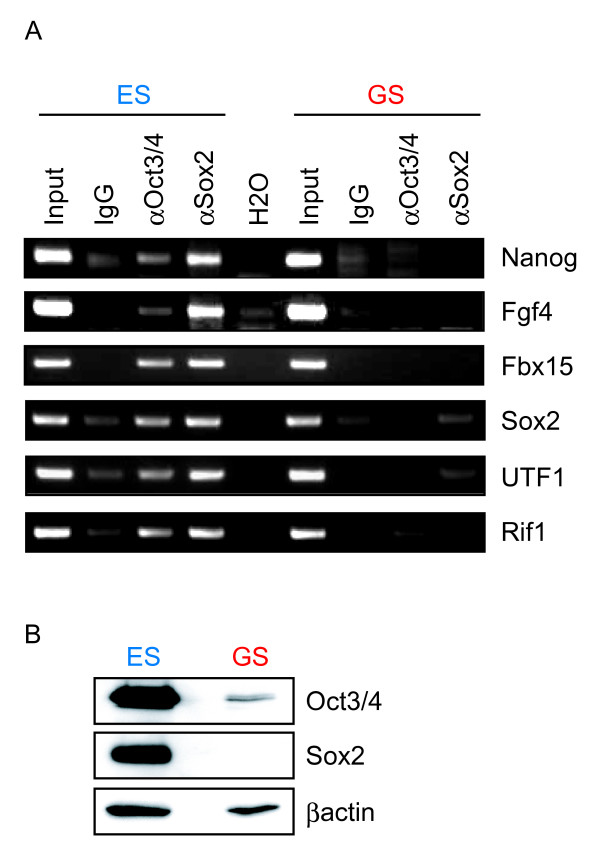
**The binding of Oct3/4 and Sox2 to the Octamer/Sox elements**. A. ChIP analyses was performed with anti-Oct3/4 antibody (αOct3/4) and anti-Sox2 antiserum (αSox2), or with normal mouse IgG as a negative control (IgG). The genomic region with the Octamer/Sox element in *Nanog*, *Fgf4*, *Fbx15*, *Sox2*, *UTF1*, and *Rif1 *gene was amplified with specific primers. B. Western blot analysis of Oct3/4 and Sox2 proteins in ES cells and GS cells. We used β-actin as a loading control.

To examine the effect of DNA hypermethylation on the binding of Oct3/4 and Sox2, we performed chromatin immunoprecipitaion. We found that the two transcription factors bound to the Octamer/Sox elements of *Nanog*, *Fgf4 *and *Fbx15 *genes in ES cells, but not in GS cells. (Fig. 10A). Unexpectedly, the two transcription factors did not bind to the Oct/Sox elements of *Sox2*, *UTF1*, and *Rif1 *genes either, despite their hypomethylation status in GS cells. Western blot analysed showed that the amount of Oct3/4 protein in GS cells was approximately 1/10 of that in ES cells (Fig. 10B). Furthermore, we could not detect Sox2 protein in GS cells despite the abundant transcripts. This result suggests that GS cells possess a mechanism that either inhibit translation or degrade the two proteins.

## Discussion

In this study, we compared the expression and DNA methylation state of ECATs in ES cells, GS cells, and somatic cells. We found that many ECATs, including *Oct3/4 *and *Sox2*, are expressed in GS cells in spite of their restricted potential. Furthermore, the regulatory regions of these genes were hypomethylated in GS cells. However, a few genes, including *Nanog*, *ECAT1*, *Fbx15*, and *Fgf4*, were not expressed in GS cells. Among these, *Nanog*, *Fbx15*, and *Fgf4 *have been shown to be direct targets of synergistic activation by Oct3/4 and Sox2. The Octamer motif and Sox-binding sites of these three genes were hypermethylated in GS cells. In addition, we found that GS cells express little Sox2 and low Oct3/4 protein in spite of the high RNA expression levels. These data indicate that repression and DNA hypermethylation of a small set of ECATs, and the post-transcriptional suppression of Oct3/4 and Sox2 may contribute to loss of pluripotency in male germ cells.

Germ cells must perform two contradictory tasks: on the one hand, they must transmit totipotency and pluripotency to the next generation. On the other hand, germ cells themselves must lose totipotency and pluripotency to avoid tumor formation, especially teratomas. Since ECATs play important roles in totipotency and pluripotency, it is likely that male germ cells maintain expression of ECATs, in part by hypomethylation, in order to retain pluripotency. In mature sperm, global transcription is suppressed by heterochromatin formation, including the displacement of histones by transition proteins and protamines[25]. Thus, most ECATs are probably not transcribed in mature sperm. Nevertheless, we found that the promoter regions of most ECATs were hypomethylated in adult testes and isolated sperm. Presumably, this allows rapid activation of ECAT expression following fertilization.

Our data showed that the binding sites of Oct3/4 and Sox2 in some genes were specifically hypermethylated in the male germline, possibly resulting in loss of pluripotency in male germ cells. Nanog may play an essential role in this process. *Nanog *expression is restricted to pluripotent cells, such as ES cells, EC cells, EG cells, and mGS cells *in vitro*, and the ICM and PGCs *in vivo*[6,7,26-28]. Loss of *Nanog *expression leads to loss of pluripotency, whereas overexpression actively promotes pluripotency[6,7]. *Nanog *expression is not observed in germ cells after they settle in the genital ridge, in either sex[28]. By contrast, some reports have indicated that germ cell tumors in human testes ectopically express *NANOG*[29,30]. It is likely that in order for pluripotent epiblast cells to committ the unipotent germline stem cell fate, target genes of Oct3/4 and Sox2 must be repressed by DNA hypermethylation. Upon fertilization, male pronucleus undergoes active DNA demethylation, and thus erases the hypermethylation of the Oct/Sox sites[31].

In addition to DNA hypermethylation in the Octamer/Sox elements, we found that Oct3/4 and Sox2 themselves are regulated at protein levels. Although Sox2 mRNA is abundant in GS cells, we could not detect Sox2 protein. Oct4 protein also showed a lower protein level than the mRNA level in GS cells. Thus the expressions of ECATs in GS cells are suppressed not only by DNA hypermethylation, but also by suppressed protein expression of Sox2 and Oct3/4.

An open question is what mechanisms lead to hypermethylation of the Octamer/Sox elements. We observed germ cell-specific hypermethylation of the Octamer/Sox element of *Fbx15*, *Fgf4*, *Nanog*, and *Oct3/4 *and of the two POU-binding sites of *Sox2 *(Fig. 4, 5, 6). However, DNA methylation was not observed for the Octamer/Sox elements of *Utf1 *and *Sox2 *or the Rox1 and Oct3/4-binding site of *Rex1 *(Fig. 2, 6). Previous studies provide some clues to help explain this discrepancy. It was shown by gel-mobility shift assay that heterodimers of Oct1 or Oct6 and Sox2 can bind *in vitro *to Oct3/4 and Sox2 binding sites in *Fgf4 *but not in *Utf1*, due to differences in the DNA sequences[11]. This suggests that differential binding stringency of Octamer/Sox elements affects the recruitment of protein complexes. Thus, properties intrinsic to individual Octamer/Sox elements might determine whether or not they are recognized by different protein complexes, including DNA methyltransferases. However, further studies are required to uncover the precise mechanism of selective hypermethylation in male germ cells.

Notably, the clustered distribution of DNA methylation in the *Nanog *locus suggests that waves of DNA methylation may spread out in both directions from "methylation centers." During tumorgenesis, an initial "seeding" methylation event is known to induce hypermethylation in CpG islands[32,33]. The "methylation center" regions in the *Nanog *locus might similarly function as physiological methylation "seeding" sites. We could not find any obvious protein binding sites commonly to these regions, but it is possible that proteins bind these regions and regulate chromosome structure and transcription. Recent studies have revealed that a nuclear structure known as a chromatin loop is associated with gene expression. SATB1, a nuclear protein that binds to AT-rich sequences, forms cage-like networks and regulates gene expression in higher order chromatin structures in lymphocytes[34]. Mecp2, which binds methylated CpG sequences, regulates the silent chromatin loop in the *Dlx5*-*Dlx6 *locus, with expression of *Dlx5 *and *Dlx6 *elevated in the brains of *Mecp2*-deficient mice[35]. These chromatin loop binding proteins are potential candidates for regulators of methylation centers.

## Conclusion

In the current study, we examined the expression and DNA methylation status of ES cell maker genes (ECAT for ES cell associated transcripts). In all ECATs examined, the promoter region had low methylation levels in ES cells, but higher levels in somatic cells. In contrast, in spite of their lack of pluripotency, male germline stem (GS) cells expressed most ECATs and exhibited hypomethylation of ECAT promoter regions. However, a few ECATs were not expressed in GS cells, and most of them targets of Oct3/4 and Sox2. The Octamer/Sox regulatory elements were hypermethylated in these genes. Our results suggest that DNA hypermethylation and transcriptional repression of a small set of ECATs might contribute to the loss of pluripotency in male germ cells.

## Methods

### Cell culture

RF8 ES cells were cultured on gelatin-coated plates in Dulbecco's modified Eagle medium (Nacalai Tesque) supplemented with 0.1 mM non-essential amino acids (GIBCO BRL), 2 mM L-glutamine (GIBCO BRL), 50 U/ml Penicillin-Streptomycin (GIBCO BRL), 0.11 mM 2-mercaptoethanol (GIBCO BRL), 15% FBS (Biowest or Hyclone), and 0.01% conditioned medium of PLAT-E cells transfected with an expression vector for leukemia inhibitory factor (LIF).

To induce cell differentiation, ES cells were seeded at a density of 2 × 10^5 ^cells per 100 mm plate and cultured in medium supplemented with LIF for 24 hours. After rinsing with PBS, the medium was changed to fresh medium supplemented with 3 × 10^-7 ^M retinoic acid (RA, Sigma) and without LIF. Medium was exchanged every 2 days.

Mouse embryonic fibroblasts (MEFs) were prepared as described previously[36]. The GS cells were established from the testes of a newborn DBA/2 mouse, and cultured as described[22].

### Preparation of genomic DNA and bisulfite genomic sequencing

To prepare genomic DNA from cultured cells, cells were washed with PBS and lysed with PUREGENE Cell Lysis Solution (GENTRA SYSTEMS) at 37°C overnight. For preparation of DNA from mouse tissues, isolated organs were rapidly frozen in liquid nitrogen and crushed with a few strokes of a hammer. Small pieces of frozen organs were transferred into lysis buffer (50 mM Tris-HCl pH8.0, 200 mM NaCl, 25 mM EDTA, 0.2% SDS, 0.1 mg/ml protease K) and were rotated overnight at 55°C. On the next day, the lysates were treated with 0.033 mg/ml RNase A (Nacali tesque) at 37°C for 5 minutes and genomic DNA was extracted by phenol-chloroform extraction and ethanol precipitation. Extracted DNA was dissolved in 10 mM Tris-HCl (pH 8.0).

Sodium bisulfite treatment of genomic DNA was performed with the CpGenome DNA Modification Kit (CHEMICON) according to the manufacturer's protocol with some modifications. Genomic DNA (1 μg in 100 μl water) was denatured by addition of 7 μl of fresh 3N NaOH, then incubated at 50°C for 10 minutes. After adding 550 μl of Reagent I solution, the DNA solution was incubated at 50°C for 16 hours. Following incubation with Reagent I, 750 μl of Reagent II solution was added and incubated at room temperature for 10 minutes. The DNA was purified using the Qiaquick gel extraction kit (Qiagen) according to the manufacturer's instructions and eluted from the kit column in 50 μl of elution buffer. The bisulfite reaction was completed by the addition of 5 μl of fresh 3N NaOH followed by 5 minutes incubation at room temperature. The DNA was purified again using the Qiaquick gel extraction kit and eluted in 30 μl of elution buffer.

For sodium bisulfite treatment of oocyte genomic DNA, genomic DNA was digested with *BamH*I, *EcoR*V, or *Spe*I, followed by phenol-chloroform extraction and ethanol precipitation. After incubation in 0.33 M NaOH at 37°C for 15 minutes, sodium metabisulfite (pH5.0, SIGMA) and hydroquinone (SIGMA) were added to final concentrations of 2.0 M and 0.5 mM, respectively. Following incubation in the dark at 55°C for 12 hours, the DNA was purified with the Wizard DNA Clean-Up system (Promega) and incubated in 0.3 M NaOH at 37°C for 15 minutes. Ammonium acetate was added at a final concentration of 3 M, and the DNA was ethanol precipitated in the presence of sodium acetate and glycogen. Purified DNA was suspended in 20 μl of 10 mM Tris-HCl (pH 8.0).

For PCR, 1 μl of DNA suspension was amplified in the first round of PCR, and 1/10 volume of the first PCR product was used as a template for the second round of PCR. The primers used for amplification of genomic fragments are described in the Additional files [see Additional file 1]. PCR products were gel purified using the Qiaquick gel extraction kit, and cloned into pCR2.1 with the TOPO TA Cloning Kit (Invitrogen). Ten random clones were picked and sequenced with M13 or M13 reverse primer. Clones with incomplete bisulfite conversion were discarded from the analysis.

### RNA isolation and reverse transcription

Total RNA was isolated from cells or adult mouse testes with TRIzol reagent (Invitrogen) according to the manufacturer's instructions. Extracted RNA was dissolved in DEPC-treated water. To eliminate contaminating genomic DNA, RNA solutions were treated with TURBO DNase (Ambion) at 37°C for 1 hour. RNA (1 μg) was reverse transcribed with ReveTra Ace (TOYOBO) in a 20 μl reaction volume using oligo-dT primer.

### RT-PCR and Real-time PCR

RT-PCR was performed with ExTaqHS (TaKaRa) in a 25 μl reaction volume. As template, 0.5 μl of cDNA was used. Specific primers and PCR conditions are described in the Additional files [see Additional file 2]. Real-time PCR was performed using the 7300 Real Time PCR System (Applied Biosystems) and Platinum SYBR Green qPCR SuperMix UDG (Invitrogen) according to the manufacturer's instructions.

### Chromatin immunoprecipitation

Approximately 2 × 10^6 ^GS cells and ES cells cultured without feeder cells were tripsinized and centrifused at 1000 rpm for 5 minutes. Cells were resuspended in 1 ml of ES medium and fixed by the addition of 27 μl of 37% formaldehyde and rotation at room temperature for 8 minutes. Cross-link reaction was stopped by the addition of 50 μl of 2.5 M Glycine. Following to rinsing with PBS three times, 200 μl of SDS Lysis Buffer (1%SDS, 10 mM EDTA, 50 mM Tris-HCl [pH8.0]) with 1x protease inhibitor (Complete, Roche) was added and incubated on ice for 10 minutes. Cell lysates were sonicated for 30 seconds at 10 times with 1 minute intervals using a Bioruptor (Cosmo Bio, Japan). After dilution with 1.8 ml of ChIP Dilution Buffer (0.01% SDS, 1.1% Triron X-100, 1.2 mM EDTA, 16.7 mM Tris-HCl [pH8.0], 167 mM NaCl), 200 μl of each cell lysates was collected as an input. Remaining cell lysates were precleared with 2 μg of normal mouse IgG in the presence of protein G-Sepharose bead slurry (60 μl of 50/50 slurry of beads in ChIP Dilution Buffer supplemented with 1 mg/ml BSA and 200 μg/ml salmon sperm DNA). Samples were rotated at 4°C for 2 hours. Unbound materials were collected by centrifugation at 15000 rpm for 1 minute, and divided into three tubes. To precipitate Oct3/4 and Sox2, 5 μg of anti-Oct3/4 antibody (C10, Santa Cruz Biotechnology) and 1 μl of anti-Sox2 antiserum [37] were added respectively, and rotated at 4°C overnight. Alternatively, 2 μg of normal rabbit IgG was added as a negative control. On the next day, 20 μl of blocked protein G slurry was added and rotated at 4°C for 2 hours. Beads were collected by centrifugation at 15000 rpm for 1 minute. Beads were washed once sequentially with 1 ml of ice-cold Low Salt immune complex wash buffer (0.1% SDS, 1% Triton X-100, 2 mM EDTA, 20 mM Tris-HCl [pH7.6], 150 mM NaCl), High Salt immune complex wash buffer (0.1% SDS, 1% Triton X-100, 2 mM EDTA, 20 mM Tris-HCl [pH8.0], 500 mM NaCl), LiCl immune complex wash buffer (0.25 M LiCl, 1% Nonidet P-40, 1% deoxycholate, 1 mM EDTA, 10 mM Tris-HCl [pH 8.0]), and then twice with TE buffer (10 mM Tris-HCl, 1 mM EDTA, pH8.0). Bound materials were eluted from the beads in 200 μl of elution buffer (1% SDS, 0.1 M NaHCO3) with 8.5 μl of 5 M NaCl by rotation at room temperature for 15 minutes. Cross-linking was reversed by incubation at 65°C overnight. Stored input was also treated for cross-link reversal with 8.5 μl of 5 M NaCl. Eluted samples were diluted with 200 μl of 10 mM Tris-HCl (pH8.0) and treated with 20 μg of RNase A at 37°C for 30 minutes. Then samples were treated with 30 μg of proteinase K at 55°C for 1 hour. Following to phonol-chloroform extraction and ethanol precipitation, purified DNA was dissolved in 30 μl of 10 mM Tris-HCl (pH8.0) for ChIP products or 50 μl of 10 mM Tris-HCl (pH8.0) for inputs. Semi-quantitative PCR was performed with ExTaqHS (Takara, Japan). Specific primers are described in the Additional files [see Additional file 3].

### Western blot analyses

Cell lysates obtained during chromatin immunoprecipitaion procedure were separated on SDS-PAGE, and transferred to PVDF membrane (Immobilon-P, Millipore Corporation). The membrane was blocked with 5% skim milk and incubated with primary antibodies in TBST (20 mM Tris-HCl (pH 8.0), 300 mM NaCl, 0.15% Tween 20) with 0.5% skim milk overnight at 4°C. Anti-bodies used were anti-Oct3/4 antibody (1:600), anti-Sox2 antiserum (1:1000), and anti-βactin antibody (1:4000, Sigma). The secondary antibodies used were anti-rabbit IgG HRP-linked Antibody (1:5000, #7074; Cell Signaling Technology) or anti-mouse IgG HRP-linked Antibody (1:3000, #7076; Cell Signaling Technology). Signal was detected with ECL Western blotting Detection Reagents (RPN2106; Amersham Biosciences) using LAS3000 (Fuji film, Japan). To reprobe, the membrane was gently shaken in prewarmed Stripping buffer (62.5 mM Tris-HCl [pH6.7], 100 mM 2-mercaptoethanol, 2% SDS) at 50°C for 20 minutes.

## Authors' contributions

MI carried out most of the experiments in this study. KM and KI helped bisulfite genomic sequencing. TI carried out mouse embryo manipulation. MN carried out ES cell culture. JL, MK-S, and TS carried out GS cell derivation and culture. SY conceived of the study, and participated in its design and coordination and helped to draft the manuscript. All authors read and approved the final manuscript.

## Supplementary Material

Additional File 1 Primers and PCR conditions for bisulfite genomic sequencing. Click here for file

Additional File 2 Primers and PCR conditions for RT-PCR. Click here for file

Additional File 3 Primers for chromatin immunoprecipitation. Click here for file
