# Supplementary material for: Transcriptional repression and DNA hypermethylation of a small set of ES cell marker genes in male germline stem cells
Source: BMC Dev Biol. 2006 Jul 21;6:34. doi: 10.1186/1471-213X-6-34 (PMC1564388; doi:10.1186/1471-213X-6-34)
Supplement: Additional File 1 — Primers and PCR conditions for bisulfite genomic sequencing. [file 1471-213X-6-34-S1.doc]

| Additional file 1 – Primers and PCR condition for bisulfite genomic sequencing | | | |  |  |
| --- | --- | --- | --- | --- | --- |
| Gene | PCR product | Primer name | Primer sequence | PCR condition | Product size |
| *Aicda* |  | meAicda-F1-S | GTGGTATTTGGGTTGGTTTTTTAGAGGAAT | 94℃,1 min.:(94℃,2 sec.:50℃,10 sec.:68℃,30 sec.)×35 cycles:68℃,5 min.:4℃ | 370 bp |
|  |  | meAicda-F1-AS | CACAAACAAATAAAACTATCTTATTACCTA |  |  |
| *Apobec1* |  | meApobec1-F1-S | TTTGAATGTTGTTTTGTGTTTTATATTTGG | 94℃,1 min.:(94℃,2 sec.:50℃,10 sec.:68℃,30 sec.)×35 cycles:68℃,5 min.:4℃ | 400 bp |
|  |  | meApobec1-F1-AS | AAAAAAAAAACGCCTTACTATTACTACCTA |  |  |
| *ECAT8* | PCR product 1 | me77010-F2-S | AATTTTTTTTGAGGGTTGGTGAGATGGTTT | 94℃,1 min.:(94℃,2 sec.:50℃,10 sec.:68℃,30 sec.)×35 cycles:68℃,5 min.:4℃ | 512 bp |
|  |  | me77010-F2-AS | ATCAACCACCCAAATTTTATATCTTCCTAA |  |  |
|  | PCR product 2 | me77010-F1-S | TATTATGTATTTTTTTTAGTTTTGTAGTTT | 94℃,1 min.:(94℃,2 sec.:50℃,10 sec.:68℃,30 sec.)×35 cycles:68℃,5 min.:4℃ | 485 bp |
|  |  | me77010-F1-AS | AAATATATTAACATTTTACCACCCCATCTA |  |  |
|  | PCR product 3 | me77010-F5-S | GATGTATTTTTTGGTTGTAGATGGGGTGGT | 94℃,1 min.:(94℃,2 sec.:50℃,10 sec.:68℃,30 sec.)×35 cycles:68℃,5 min.:4℃ | 350 bp |
|  |  | me77010-F5-AS | TCAATCCTCAAATCTATTACCCTCTACATA |  |  |
| *ECAT15-2* |  | meECAT15-2-S | ATATAGAAGGAGTGGAAAGGTTTGTTTTTT | 94℃,1 min.:(94℃,2 sec.:50℃,10 sec.:68℃,30 sec.)×35 cycles:68℃,5 min.:4℃ | 480 bp |
|  |  | meECAT15-2-AS | TACTTCCTATCTTCCTATAACCCAATTAAA |  |  |
| *ERas* |  | meERas-F3-S | TTTTTGTTGTTTTTGGGGGTAGGGAGTATT | 95℃,1 min.:(95℃,10 sec.:52℃,10 sec.:72℃,30 sec.)×35 cycles:72℃,5 min.:4℃ | 446 bp |
|  |  | meERas-F3-AS | TCCAAATAAAAAACCCTATAACTTCCTTAA |  |  |
| *Esg1* |  | meESG1-F1-S | GATGGGAAAAGTTATAGTTTTTTAGAAGTT | 94℃,1 min.:(94℃,2 sec.:50℃,10 sec.:68℃,30 sec.)×35 cycles:68℃,5 min.:4℃ | 484 bp |
|  |  | meESG1-F1-AS | AAATACTTCTAAATCTTTCAAATCTTCAAA |  |  |
| *Fbx15* | PCR product 1 | fbx.pro-scU488 | TTTGATTTAAATTATAGGTATGATTAATTT | 94℃,1 min.:(94℃,2 sec.:50℃,10 sec.:68℃,30 sec.)×35 cycles:68℃,5 min.:4℃ | 168 bp (Nested PCR) |
|  |  | fbx.pro-scL712 | ACTAACTAACTAATTAATCCACCTTAACAA |  |  |
|  |  | fbx.pro-scU524 | AGGAGAAGATATTAAGTAGAAATTTTTTTT | 94℃,1 min.:(94℃,2 sec.:50℃,10 sec.:68℃,30 sec.)×35 cycles:68℃,5 min.:4℃ |  |
|  |  | fbx.pro-acL692 | ACCTTAACAATTTATCTAACAAACCCCTAA |  |  |
|  | PCR product 2 | mFBXpro-Sc-U848 | ATATTTAGGGTTTGAGTAAGGGTGGTGTAATG | 94℃,1 min.:(94℃,2 sec.:50℃,10 sec.:68℃,30 sec.)×35 cycles:68℃,5 min.:4℃ | 436 bp |
|  |  | mFBXpro-Sc-L1253 | TCCCACGAAACTATTTATTTCAACTAATCTTC |  |  |
|  | PCR product 3 | meFbx15(Testis)-F1-S | TAAACGTAGTTGAATAGGGTTATAAAAGGT | 94℃,1 min.:(94℃,2 sec.:50℃,10 sec.:68℃,30 sec.)×35 cycles:68℃,5 min.:4℃ | 506 bp |
|  |  | meFbx15(Testis)-F1-AS | ATATAAAAATTTCCCTTTACCATTCTTAAA |  |  |
| *Fgf4* | PCR product 1 | meFGF4-F2-S | TATATTTTGATAGTTAAGTTATAGTGTGAT | 94℃,1 min.:(94℃,2 sec.:50℃,10 sec.:68℃,30 sec.)×35 cycles:68℃,5 min.:4℃ | 289 bp |
|  |  | meFGF4-F2-AS | TCGCCTAAAAACCGAAACGCAAACCGCCTA |  |  |
|  | PCR product 2 | meFGF4-F1-S | TTTTAGGTAGTTTTTATGTAGGTAGATTGT | 94℃,1 min.:(94℃,2 sec.:50℃,10 sec.:68℃,40 sec.)×35 cycles:68℃,5 min.:4℃ | 550 bp |
|  |  | meFGF4-F1-AS | TTCCAATCTTACTATCTATAACCTCCCATA |  |  |
|  | PCR product 3 | meFGF4-F3-S | TTTAGGTTTTAAGAGTGTTGGGGAGAAGAT | 94℃,1 min.:(94℃,2 sec.:50℃,10 sec.:68℃,40 sec.)×35 cycles:68℃,5 min.:4℃ | 517 bp |
|  |  | meFGF4-F3-AS | TACAAAACAAAAACATCAAACCCATTCTAA |  |  |
| *Gdf3* |  | meGDF3-F1-S | TTAATGTTTGTGTGTTGAATGAGTGTGTTT | 94℃,1 min.:(94℃,2 sec.:57℃,10 sec.:72℃,30 sec.)×35 cycles:72℃,5 min.:4℃ | 584 bp |
|  |  | meGDF3-F1-AS | CCACAACACCCACTTAAAATTTTTCTCTAA |  |  |
| *LOC384480* |  | meLOC384480-F1-S | GTTGTTTTGGTTATGGTGTTTGTTTATAGT | 94℃,1 min.:(94℃,2 sec.:51℃,10 sec.:68℃,40 sec.)×35 cycles:68℃,5 min.:4℃ | 374 bp |
|  |  | meLOC384480-F1-AS | TACAACTACATCAACTACTCATAAAACATA |  |  |
| *LOC626074* |  | meLOC434084-F1-S | GGTTGGGCGTGGTAGGTATATTTTTAAGTT | 94℃,1 min.:(94℃,2 sec.:51℃,10 sec.:68℃,40 sec.)×35 cycles:68℃,5 min.:4℃ | 501 bp |
|  |  | meLOC434084-F1-AS | CGAAAACCACCACGACCTCCTAATCCTAAA |  |  |
| *Mvh* |  | meMvh-F1-S | AGGTTTTATAGGTTATGGAGTTAAGAGGTT | 94℃,1 min.:(94℃,2 sec.:52℃,10 sec.:72℃,30 sec.)×35 cycles:72℃,5 min.:4℃ | 283 bp |
|  |  | meMvh-F1-AS | CCGCCGACCCACTCACCTCTCCGCTCCAAA |  |  |
| *Nanog* | PCR product 1 | meNanog-F3-S | GGAATGTAGTAAGTTTGTTTTTTGGTTATT | 94℃,1 min.:(94℃,2 sec.:50℃,10 sec.:68℃,40 sec.)×35 cycles:68℃,5 min.:4℃ | 434 bp |
|  |  | meNanog-F3-AS | AAAAAATACCACCAAAAAACCCTAAATATA |  |  |
|  | PCR product 2 | meNanog-F15-S | TTAGAGTTTGAATTAGTTAGTTTTTTGGAT | 94℃,1 min.:(94℃,2 sec.:50℃,10 sec.:68℃,30 sec.)×35 cycles:68℃,5 min.:4℃ | 491 bp |
|  |  | meNanog-F15-AS | TATAATAACTCTTATCTCCCCATTCCTAAA |  |  |
|  | PCR product 3 | meNanog-F13-S | TGAGATTGGAGTAGAGGGTGGGAAAGGATT | 94℃,1 min.:(94℃,2 sec.:52℃,10 sec.:68℃,30 sec.)×35 cycles:68℃,5 min.:4℃ | 467 bp |
|  |  | meNanog-F13-AS | TACAAACCAACCTAACTTTAAACTCCCAAA |  |  |
|  | PCR product 4 | meNanog-F12-S | GTTATAGATAATATTGTAGTTTTTGGTTAG | 94℃,1 min.:(94℃,2 sec.:50℃,10 sec.:68℃,30 sec.)×35 cycles:68℃,5 min.:4℃ | 310 bp (Nested PCR) |
|  |  | meNanog-F12-AS | TCCTAAAATTAAATCTATAAACCAAACTAA |  |  |
|  |  | meNanog-F12-AS2 | AAAACAAAAAAATAACTATTCTACCTTCTA | 94℃,1 min.:(94℃,2 sec.:50℃,10 sec.:68℃,30 sec.)×35 cycles:68℃,5 min.:4℃ |  |
|  | PCR product 5 | meNanog-F11-S | AGAGGTTTAGTTAGGTTGGGTAATGGAGGT | 95℃,1 min.:(95℃,10 sec.:58℃,10 sec.:72℃,30 sec.)×35 cycles:72℃,5 min.:4℃ | 456 bp |
|  |  | meNanog-F11-AS | CAATACCACCACTATACCACAACACCACTA |  |  |
|  | PCR product 6 | meNanog-F4-S | GTTTTTTGTAGAATAAAATTTAGGAAGAAT | 94℃,1 min.:(94℃,2 sec.:51℃,10 sec.:68℃,30 sec.)×35 cycles:68℃,5 min.:4℃ | 378 bp |
|  |  | meNanog-F4-AS | CTAACTTCAAACTTACTACAATCCCAAATA |  |  |
|  | PCR product 7 | meNanog-F1-S | GTTATTTAAGGTAATAGAGAAAAATTTGTT | 94℃,1 min.:(94℃,2 sec.:50℃,10 sec.:68℃,30 sec.)×35 cycles:68℃,5 min.:4℃ | 459 bp |
|  |  | meNanog-F1-AS | ACAAAAAAAACTATAAAATAACCCAAACTA |  |  |
|  | PCR product 8 | meNanog-F2-S | GATTTTGTAGGTGGGATTAATTGTGAATTT | 95℃,1 min.:(95℃,10 sec.:52℃,10 sec.:72℃,30 sec.)×35 cycles:72℃,5 min.:4℃ | 367 bp |
|  |  | meNanog-F2-AS | ACCAAAAAAACCCACACTCATATCAATATA |  |  |
|  | PCR product 9 | meNanog-F5-S | TTTTTATAGTTTGTTTAGTTTTGAGGAAGT | 94℃,1 min.:(94℃,2 sec.:50℃,10 sec.:68℃,30 sec.)×35 cycles:68℃,5 min.:4℃ | 452 bp |
|  |  | meNanog-F5-AS | CATTCCAAACTAAAATATTAAATCTCCCTA |  |  |
|  | PCR product 10 | meNanog-F7-S | TGGTGAGGTTATATAGTTAGTTTGTAATTT | 94℃,1 min.:(94℃,2 sec.:50℃,10 sec.:68℃,40 sec.)×35 cycles:68℃,5 min.:4℃ | 420 bp |
|  |  | meNanog-F10-AS | ACTCAAAAAAAAAAAAACACACACTTCCTA |  |  |
|  | PCR product 11 | meNanog-F16-S | TATGTGGTTGTTGGGAATTGAATTTAGGAT | 94℃,1 min.:(94℃,5 sec.:57℃,10 sec.:72℃,40 sec.)×35 cycles:72℃,5 min.:4℃ | 531 bp |
|  |  | meNanog-F16-AS | CAAAACCAACCAAACAAATAAATCATTTTC |  |  |
| *Nmyc1* |  | meNmyc1-F1-S | TTAGAGAGTTAGTATTTTGAGGGAGAATTT | 94℃,1 min.:(94℃,2 sec.:50℃,10 sec.:68℃,30 sec.)×35 cycles:68℃,5 min.:4℃ | 411 bp |
|  |  | meNmyc1-F1-AS | AAACCCTTTTTCAACACACATTCTCAATAA |  |  |
| *Oct3/4* | PCR product 1 | meOct3-F2-S | TGTTGTTAAGGATTGTATTATATTTTAGGT | 94℃,1 min.:(94℃,2 sec.:50℃,10 sec.:68℃,40 sec.)×35 cycles:68℃,5 min.:4℃ | 505 bp |
|  |  | meOct3-F2-AS | CTATACATTCATTATAAAACAATACCATAA |  |  |
|  | PCR product 2 | Oct PRO F | TGGGTTGAAATATTGGGTTTATTT | 94℃,1 min.:(94℃,2 sec.:54℃,10 sec.:68℃,40 sec.)×35 cycles:68℃,5 min.:4℃ | 533 bp |
|  |  | Oct PRO R | CTAAAACCAAATATCCAACCATA |  |  |
| *Preproacrosin* |  | meAcrosin-F1-S | AAAATGGTTTTTTGAAAGTGTTTTGGTGAT | 94℃,1 min.:(94℃,5 sec.:54℃,10 sec.:72℃,30 sec.)×35 cycles:72℃,5 min.:4℃ | 399 bp |
|  |  | meAcrosin-F1-AS | CACCCCAACAATTAAACCAAACAAACCTCC |  |  |
| *REST*/*NRSF* |  | meREST-F1-S | GGATAATAGTTTTTGTAAATGTTAAATGAA | 94℃,1 min.:(94℃,2 sec.:50℃,10 sec.:68℃,30 sec.)×35 cycles:68℃,5 min.:4℃ | 286 bp |
|  |  | meREST-F1-AS | AATCTACACAACAAATTCTAAACCTACCAA |  |  |
| *Rex1* | PCR product 1 | meRex1-F2-S | TTTATTTTTATTTAGAGGAATGAGAGATAT | 94℃,1 min.:(94℃,2 sec.:50℃,10 sec.:68℃,30 sec.)×35 cycles:68℃,5 min.:4℃ | 361 bp |
|  |  | meRex1-F2-AS | ATTACCTAAACTTTTTCCAACATTAATATA |  |  |
|  | PCR product 2 | meRex1-F1-S | TATATTAATGTTGGAAAAAGTTTAGGTAAT | 94℃,1 min.:(94℃,2 sec.:50℃,10 sec.:68℃,30 sec.)×35 cycles:68℃,5 min.:4℃ | 498 bp |
|  |  | meRex1-F1-AS | AACTCCTTAAACCCCTCCCTTTTTAAATAA |  |  |
| *Rif1* |  | meRif1-F1-S | TGTTTGTTTAGTTAGTTTAAGTGTTGGGAG | 94℃,1 min.:(94℃,5 sec.:52℃,10 sec.:72℃,30 sec.)×35 cycles:72℃,5 min.:4℃ | 415 bp |
|  |  | meRif1-F1-AS | CTTCTAAAAATAAAAACTAATAACAACACA |  |  |
| *Slc2a3* |  | meSlc2a3-F1-S | AAGAATTACGAGGAGGATGTGGTAAAAAGT | 94℃,1 min.:(94℃,2 sec.:51℃,10 sec.:68℃,40 sec.)×35 cycles:68℃,5 min.:4℃ | 314 bp |
|  |  | meSlc2a3-F1-AS | CTTAAAATAAATCTAAAAATATCCAACCAA |  |  |
| *Sox2* | PCR product 1 | meSox2-F3-S | GTTTTAATTGTTAAATAGGGTTTTTTTTAG | 94℃,1 min.:(94℃,2 sec.:50℃,10 sec.:68℃,40 sec.)×35 cycles:68℃,5 min.:4℃ | 498 bp |
|  |  | meSox2-F3-AS | ACTATTAACCCAAACCAAAAACCTTAACTA |  |  |
|  | PCR product 2 | meSox2-F1-S | TTTGTTAGTTTTTTGAAATATTAGTTGGAT | 94℃,1 min.:(94℃,2 sec.:54℃,10 sec.:68℃,40 sec.)×35 cycles:68℃,5 min.:4℃ | 497 bp |
|  |  | meSox2-F1-AS | TCTCTTCTCTACCTTAACAACTCCTAATAC |  |  |
|  | PCR product 3 | meSox2-F2-S | ATTTTAGGTGTAGAGTTGGTTGTTTGATTT | 94℃,1 min.:(94℃,2 sec.:50℃,10 sec.:68℃,40 sec.)×35 cycles:68℃,5 min.:4℃ | 421 bp |
|  |  | meSox2-F2-AS | AATATACCAAACCCCTTCCTTATTCCTTTA |  |  |
| *Stella* |  | meStella-F1-S | AAATTAAAAGAGATTTTAGATAAAATTTGT | 94℃,1 min.:(94℃,2 sec.:50℃,10 sec.:68℃,30 sec.)×35 cycles:68℃,5 min.:4℃ | 414 bp |
|  |  | meStella-F1-AS | CTACCCCCAAACTACTTTAATACTAATAAA |  |  |
| *Tcf3* |  | meTcf3-F1-S | TGGGTTTTAAATTTTTTTGAGATGGTTTGG | 95℃,1 min.:(95℃,5 sec.:56℃,10 sec.:72℃,30 sec.)×35 cycles:72℃,5 min.:4℃ | 491 bp |
|  |  | meTcf3-F1-AS | GAACTCAATTCCCTCAAAAATACACTCCTA |  |  |
| *UTF1* | PCR product 1 | meUTF1-F2-S2 | AGAATAAGTAAGGTATAGGTTAAGAGGAAT | 94℃,1 min.:(94℃,5 sec.:55℃,10 sec.:72℃,30 sec.)×35 cycles:72℃,5 min.:4℃ | 165 bp |
|  |  | meUTF1-F2-AS | CGTAACCTCACCAAAAAAAAAAACTCCTCC |  |  |
|  | PCR product 2 | meUTF1-F1-S | ATATAGTTGTTATTTGATGTTTGGGGTATT | 94℃,1 min.:(94℃,2 sec.:50℃,10 sec.:68℃,40 sec.)×35 cycles:68℃,5 min.:4℃ | 551 bp |
|  |  | meUTF1-F1-AS | AACTTCCCTTAACCAAAACTTAATCTTTAA |  |  |
