# Supplementary material for: Transcriptional repression and DNA hypermethylation of a small set of ES cell marker genes in male germline stem cells
Source: BMC Dev Biol. 2006 Jul 21;6:34. doi: 10.1186/1471-213X-6-34 (PMC1564388; doi:10.1186/1471-213X-6-34)
Supplement: Additional File 2 — Primers and PCR conditions for RT-PCR. [file 1471-213X-6-34-S2.doc]

| Additional file 2 – Primers and PCR conditions for RT-PCR | | | | | | | | | |
| --- | --- | --- | --- | --- | --- | --- | --- | --- | --- |
| Gene | Primer | Primer sequence | PCR condition | | | | | | PCR product size |
|  |  |  |  | Denature | Annealing | Extension |  |  |  |
| ES cell-specific genes |  | | | | | | | | |
| *ECAT1* | RT-S (exon 2) | TGTGGGGCCCTGAAAGGCGAGCTGAGAT | 94℃,1 min. | 95℃,10 sec. | 70℃,30 sec. | | 70℃,1 min. | 4℃,foever | 164 bp |
|  | RT-AS (exon 3) | ATGGGCCGCCATACGACGACGCTCAACT |  |  |  |  |  |  |  |
| *Esg1*/*Dppa5*/*ECAT2* | ESG1-RT-S | ATAAGCTTGATCTCGTCTTCC | 94℃,1 min. | 94℃,2 sec. | 54℃,10 sec. | 72℃,40 sec. | 72℃,5 min. | 4℃,foever | 501 bp |
|  | ESG1-RT-AS | CTTGCTAGGATGTAACAAAGC |  |  |  |  |  |  |  |
| *Fbx15*/*ECAT3* (Common seq.) | Fbx U1110 | CGACATTCATGGCAGTAAGACT | 94℃,1 min. | 94℃,2 sec. | 62℃,10 sec. | 72℃,40 sec. | 72℃,5 min. | 4℃,foever | 320 bp |
|  | Fbx L1431 | TCGAGTACATGGACAGCGTA |  |  |  |  |  |  |  |
| *Fbx15*/*ECAT3* (ES variant) | FbxU69 | TAGATTCTTGGACTTCCGTTCA | 94℃,1 min. | 94℃,2 sec. | 55℃,10 sec. | 72℃,30 sec. | 72℃,5 min. | 4℃,foever | 220 bp |
|  | FbxL289 | ACCAAGGTCACCGCATCCAA |  |  |  |  |  |  |  |
| *Fbx15*/*ECAT3* (Testis variant) | Fbx15(Testis)U38 | TCGGATGCTGAGGGGATGTT | 95℃,1 min. | 95℃,10 sec. | 62℃,10 sec. | 72℃,30 sec. | 72℃,5 min. | 4℃,foever | 277 bp |
|  | Fbx15L289 | ACCAAGGTCACCGCATCCAA |  |  |  |  |  |  |  |
| *Nanog*/*ECAT4* | 6047 S4 | AGGGTCTGCTACTGAGATGCTCTG | 94℃,1 min. | 94℃,2sec. | 70℃,50 sec. | | 70℃,5 min. | 4℃,foever | 364 bp |
|  | 6047 AS5 | CAACCACTGGTTTTTCTGCCACCG |  |  |  |  |  |  |  |
| *ERas*/*ECAT5* | 45328 S118 | ACTGCCCCTCATCAGACTGCTACT | 94℃,1 min. | 94℃,2sec. | 70℃,50 sec. | | 70℃,5 min. | 4℃,foever | 210 bp |
|  | ERas AS304 | CACTGCCTTGTACTCGGGTAGCTG |  |  |  |  |  |  |  |
| *Dnmt3L*/*ECAT7* | Dnmt3L-type-S | CATCTGTGAGAGCCCCGACTG | 94℃,1 min. | 94℃,2 sec. | 55℃,10 sec. | 72℃,40 sec. | 72℃,5 min. | 4℃,foever | 504 bp |
|  | Dnmt3L-1061-1042 | GGCAGCGCATACTGCAGGAT |  |  |  |  |  |  |  |
| *ECAT8* | m77010 U362 | CTGGTGCAGGGCTCTGATTAAGTC | 94℃,1 min. | 94℃,2 sec. | 62℃,10 sec. | 72℃,40 sec. | 72℃,5 min. | 4℃,foever | 786 bp |
|  | m77010 L1148 | TTTACACAGCCGTTCTTCTCGTCA |  |  |  |  |  |  |  |
| *Gdf3*/*ECAT9* | GDF3 U253 | GTTCCAACCTGTGCCTCGCGTCTT | 94℃,1 min. | 94℃,2 sec. | 62℃,10 sec. | 72℃,40 sec. | 72℃,5 min. | 4℃,foever | 570 bp |
|  | GDF3 L16914 | AGCGAGGCATGGAGAGAGCGGAGCAG |  |  |  |  |  |  |  |
| *Sox15*/*ECAT10* | Sox15-gw-s | CACCATGGCGCTGACCAGCTCCTCACAA | 94℃,1 min. | 94℃,2 sec. | 60℃,10 sec. | 72℃,1 min. | 72℃,5 min. | 4℃,foever | 700 bp |
|  | anti-sox15-as | TTAAAGGTGGGTTACTGGCAT |  |  |  |  |  |  |  |
| *ECAT15-1*/*Dppa4* | ECAT15-gw-S | CACCATGGAGACTGCTGGAGACAAGAAG | 94℃,1 min. | 94℃,2 sec. | 60℃,10 sec. | 72℃,30 sec. | 72℃,5 min. | 4℃,foever | 482 bp |
|  | ECAT15-1-gw-as2 | GGACCTATTCCAGAGGAACTGTCAC |  |  |  |  |  |  |  |
| *ECAT15-2*/*Dppa2* | ECAT15-2-gw-s | CACCATGTCATACTTCGGCCTGGAGACT | 94℃,1 min. | 94℃,2 sec. | 60℃,10 sec. | 72℃,30 sec. | 72℃,5 min. | 4℃,foever | 493 bp |
|  | ECAT15-2-gw-as2 | ACTCTACTCTTTTCTCCTTTGGCACCC |  |  |  |  |  |  |  |
| *Rnf17*/*ECAT16* | Rnf17L-RT-S | GACCGGGCTGGCTTCCTGTCACCTAGT | 94℃,1 min. | 94℃,10 sec. | 70℃,30 sec. | | 70℃,5 min. | 4℃,foever | 297 bp |
|  | Rnf17L-RT-AS | TTTACCATTTTCGGTGGCAAGGCTTCC |  |  |  |  |  |  |  |
| *Sall4*/*ECAT24* | Sall4 1758S | CCGAGACCCTGAAATTGCAGCAACTA | 94℃,1 min. | 94℃,2 sec. | 62℃,10 sec. | 72℃,1 min. | 72℃,5 min. | 4℃,foever | 1014 bp |
|  | Sall4 2746AS | ACGAGAAGTTCTTTCCACACCGTGTG |  |  |  |  |  |  |  |
| *Fgf4* | FGF4-RT-S | CGTGGTGAGＣATＣTTCGGAGTGG | 94℃,1 min. | 94℃,2 sec. | 68℃,30 sec. | | 68℃,5 min. | 4℃,foever | 197 bp |
|  | FGF4-RT-AS | CCTTCTTGGTCCGCCCGTTCTTA |  |  |  |  |  |  |  |
| *Oct3/4* | Oct3 U474 | CTGAGGGCCAGGCAGGAGCACGAG | 94℃,1 min. | 94℃,2 sec. | 62℃,10 sec. | 72℃,40 sec. | 72℃,5 min. | 4℃,foever | 451 bp |
|  | Oct3 L935 | CTGTAGGGAGGGCTTCGGGCACTT |  |  |  |  |  |  |  |
| *Rex1* | Rex1-F | CACCGACAACATGAATGAACAAAAA | 94℃,1 min. | 94℃,2 sec. | 53℃,10 sec. | 72℃,40 sec. | 72℃,5 min. | 4℃,foever | 893 bp |
|  | Rex1-R | CAATCTGTCTCCACCTTCAGCATTT |  |  |  |  |  |  |  |
| *Sall1* | Sall1U1 | CACCATGTCACGGAGGAAGCAAGCGAAGC | 94℃,1 min. | 94℃,2 sec. | 62℃,10 sec. | 72℃,1 min. | 72℃,5 min. | 4℃,foever | 856 bp |
|  | Sall1L825TAA | TTACAAGGGGTTGGCAGATGTTCGTAAA |  |  |  |  |  |  |  |
| *Sox2* | Sox2-RT-S | TAGAGCTAGACTCCGGGCGATGA | 94℃,1 min. | 94℃,2 sec. | 56℃,10 sec. | 72℃,30 sec. | 72℃,5 min. | 4℃,foever | 296 bp |
|  | Sox2-RT-AS | TTGCCTTAAACAAGACCACGAAA |  |  |  |  |  |  |  |
| *Stella*/*Dppa3* | stella ORF-S | CACCATGGAGGAACCATCAGAGAAAGTC | 94℃,1 min. | 94℃,2 sec. | 60℃,10 sec. | 72℃,30 sec. | 72℃,5 min. | 4℃,foever | 457 bp |
|  | stella ORF-AS | CTAATTCTTCCCGATTTTCGCATTCT |  |  |  |  |  |  |  |
| *Tcl1* | tcl1 ORF-S | CACCATGGCTACCCAGCGGGCACACA | 94℃,1 min. | 94℃,2 sec. | 62℃,10 sec. | 72℃,30 sec. | 72℃,5 min. | 4℃,foever | 358 bp |
|  | tcl1 ORF-AS2 | TTATTCATCGTTGGACTCCGAGTCTATCAG |  |  |  |  |  |  |  |
| *UTF1* | UTF1-RT-S | GGATGTCCCGGTGACTACGTCTG | 95℃,1 min. | 95℃,5 sec. | 63℃,10 sec. | 72℃,30 sec. | 72℃,5 min. | 4℃,foever | 344 bp |
|  | UTF1-RT-AS | GGCGGATCTGGTTATCGAAGGGT |  |  |  |  |  |  |  |
| Loading conrol |  | | | | | | | | |
| *NAT1* | NAT1 U283 | ATTCTTCGTTGTCAAGCCGCCAAAGTGGAG | 94℃,1 min. | 94℃,2 sec. | 60℃,10 sec. | 72℃,30 sec. | 72℃,5 min. | 4℃,foever | 223 bp |
|  | NAT1 L476 | AGTTGTTTGCTGCGGAGTTGTCATCTCGTC |  |  |  |  |  |  |  |
| *Gapdh* | G3PDH control primer F | ACCACAGTCCATGCCATCAC | 94℃,1 min. | 94℃,2 sec. | 55℃,10 sec. | 72℃,30 sec. | 72℃,5 min. | 4℃,foever | 450 bp |
|  | G3PDH control primer R | TCCACCACCCTGTTGCTGTA |  |  |  |  |  |  |  |
| Germ cell-specific genes |  | | | | | | | | |
| *Mvh* | Mvh Vas 3 | TTGGTTGATCAGTTCTCGAG | 94℃,1 min. | 94℃,2 sec. | 58℃,10 sec. | 72℃,30 sec. | 72℃,5 min. | 4℃,foever | 450 bp |
|  | Mvh vg 2 | CCAAAAGTGACATATATACCC |  |  |  |  |  |  |  |
| non-ECAT target of Oct3/4 and Sox2 |  |  |  |  |  |  |  |  |  |
| *REST/NRSF* | REST-RT-S | AAGAAGACGCCGCCCAAGACAAAG | 94℃,1 min. | 94℃,5 sec. | 68℃,30 sec. | | 68℃,5 min. | 4℃,foever | 218 bp |
|  | REST-RT-AS | ACCTCCATCTGGGCGGGCTCTGA |  |  |  |  |  |  |  |
| *Rif1* | Rif1-RT-S | TGATAGGCGTTGCTCTGTTGTTAG | 94℃,1 min. | 94℃,5 sec. | 54℃,10 sec. | 72℃,30 sec. | 72℃,5 min. | 4℃,foever | 295 bp |
|  | Rif1-RT-AS | ACCTCTTGCCCACATATTTGATG |  |  |  |  |  |  |  |
| *Tcf3* | Tcf3-RT-S | CGGGACAACTATGGGAAGAAGAAG | 94℃,1 min. | 94℃,5 sec. | 62℃,10 sec. | 72℃,30 sec. | 72℃,5 min. | 4℃,foever | 323 bp |
|  | Tcf3-RT-AS | AGGAAGGCAGCGGAGTGGAG |  |  |  |  |  |  |  |
| Genes neighboring the *Nanog* gene |  | | | | | | | | |
| *Aicda* | Aicda-RT-S2 | GATGGATGCCAACACGGTTAAACA | 94℃,1 min. | 94℃,5 sec. | 56℃,10 sec. | 72℃,30 sec. | 72℃,5 min. | 4℃,foever | 285 bp |
|  | Aicda-RT-AS2 | AAGCGTCATTTCCTTGCCACGGTC |  |  |  |  |  |  |  |
| *Apobec1* | Apobec1-RT-S | CATCGCAGCAACATAAGCTCC | 94℃,1 min. | 94℃,5 sec. | 65℃,10 sec. | 72℃,30 sec. | 72℃,5 min. | 4℃,foever | 487 bp |
|  | Apobec1-RT-AS | CTCTGTCATGATCTGGATAGTCACAC |  |  |  |  |  |  |  |
| *Slc2a3* | Slc2a3-RT-S | TCTCTGGTGTTCGCCGTGACTGTT | 94℃,1 min. | 94℃,2 sec. | 60℃,10 sec. | 72℃,30 sec. | 72℃,5 min. | 4℃,foever | 301 bp |
|  | Slc2a3-RT-AS | CCATAAGGCAGCCCGCAATGAT |  |  |  |  |  |  |  |
|  | | | | | | | | | |
| Real-time PCR |  | | | | | | | | |
| Gene | Primer | Primer sequence | PCR condition | | | | | |  |
|  |  |  |  |  | Denature | Anealing | Extension |  |  |
| *Esg1*/*Dppa5*/*ECAT2* | ESG1-RT-S | ATAAGCTTGATCTCGTCTTCC | 50℃,2 min. | 95℃,2 min. | 95℃,15 sec. | 55℃,30 sec. | 72℃,35 sec. | Dissociation step |  |
|  | ESG1-RT-AS | CTTGCTAGGATGTAACAAAGC |  |  |  |  |  |  |  |
| *Fbx15*/*ECAT3* (ES variant) | FbxU69 | TAGATTCTTGGACTTCCGTTCA | 50℃,2 min. | 95℃,2 min. | 95℃,15 sec. | 55℃,30 sec. | 72℃,35 sec. | Dissociation step |  |
|  | FbxL289 | ACCAAGGTCACCGCATCCAA |  |  |  |  |  |  |  |
| *Fgf4* | FGF4-RT-S | CGTGGTGAGCATCTTCGGAGTGG | 50℃,2 min. | 95℃,2 min. | 95℃,15 sec. | 68℃,30 sec. | | Dissociation step |  |
|  | FGF4-RT-AS | CCTTCTTGGTCCGCCCGTTCTTA |  |  |  |  |  |  |  |
| *GAPDH* | G3PDH control primer F | ACCACAGTCCATGCCATCAC | 50℃,2 min. | 95℃,2 min. | 95℃,15 sec. | 55℃,30 sec. | 72℃,30 sec. | Dissociation step |  |
|  | G3PDH control primer R | TCCACCACCCTGTTGCTGTA |  |  |  |  |  |  |  |
| *Nanog*/*ECAT4* | 6047 S4 | AGGGTCTGCTACTGAGATGCTCTG | 50℃,2 min. | 95℃,2 min. | 95℃,15 sec. | 70℃,50 sec. | | Dissociation step |  |
|  | 6047 AS9 | TAGCTCAGGTTCAGAATGGAGGAGAG |  |  |  |  |  |  |  |
| *Oct3/4* | Oct3 U474 | CTGAGGGCCAGGCAGGAGCACGAG | 50℃,2 min. | 95℃,2 min. | 95℃,15 sec. | 56℃,30 sec. | 72℃,40 sec. | Dissociation step |  |
|  | Oct3 L935 | CTGTAGGGAGGGCTTCGGGCACTT |  |  |  |  |  |  |  |
| *Sox2* | Sox2-RT-S | TAGAGCTAGACTCCGGGCGATGA | 50℃,2 min. | 95℃,2 min. | 95℃,15 sec. | 56℃,30 sec. | 72℃,30 sec. | Dissociation step |  |
|  | Sox2-RT-AS | TTGCCTTAAACAAGACCACGAAA |  |  |  |  |  |  |  |
| *UTF1* | UTF1-RT-S | GGATGTCCCGGTGACTACGTCTG | 50℃,2 min. | 95℃,2 min. | 95℃,15 sec. | 63℃,30 sec. | 72℃,30 sec. | Dissociation step |  |
|  | UTF1-RT-AS | GGCGGATCTGGTTATCGAAGGGT |  |  |  |  |  |  |  |
