# Supplementary material for: Transcriptional repression and DNA hypermethylation of a small set of ES cell marker genes in male germline stem cells
Source: BMC Dev Biol. 2006 Jul 21;6:34. doi: 10.1186/1471-213X-6-34 (PMC1564388; doi:10.1186/1471-213X-6-34)
Supplement: Additional File 3 — Primers for chromatin immunoprecipitation. [file 1471-213X-6-34-S3.doc]

| Additional file 3 – Primers for chromatin immunoprecipitaion |  | | |
| --- | --- | --- | --- |
| Amplified loci | Primer | Primer sequence | PCR product size |
|  |  |  |  |
| *Fbx15* | Fbx15-ChIP-S | GGATACAGTTTGACTTATTGCACG | 170 bp |
|  | Fbx15-ChIP-AS | TAGCTAGCTGGTTGGTCCACCTTA |  |
| *Fgf4* | Fgf4-ChIP-S | GCCACCAGACAGAAAGGAAGTTGGGAGG | 184 bp |
|  | Fgf4-ChIP-AS | CAGCAAGACTGGAAAATCTCATTGGCAT |  |
| *Nanog* | Nanog exonChIP-S | TCTTTAGATCAGAGGATGCCCCCTAAGC | 189 bp |
|  | Nanog exonChIP-AS | AAGCCTCCTACCCTACCCACCCCCTAT |  |
| *Rif1* | Rif1-ChIP-S | GTATCCCAGGGCCACCGTCAC | 168 bp |
|  | Rif1-ChIP-AS | AAAGGAGACCCATGGCGTGCATAAC |  |
| *Sox2* | Sox2-OctSox-S | GGGTAAGGTACTGGGAAGGGACATT | 158 bp |
|  | Sox2-OctSox-AS | ACTGTCGACTGTGCTCATTACCACG |  |
| *UTF1* | UTF1-OctSox-S | GGGAGGGCTTAGGTGCAGGTAGA | 162 bp |
|  | UTF1-OctSox-AS | CTCCTCAGGACTTCCCTTAGCCAAG |  |
